# Genetic susceptibility variants for lung cancer: replication study and assessment as expression quantitative trait loci

**DOI:** 10.1038/srep42185

**Published:** 2017-02-09

**Authors:** Giulia Pintarelli, Chiara Elisabetta Cotroneo, Sara Noci, Matteo Dugo, Antonella Galvan, Simona Delli Carpini, Lorena Citterio, Paolo Manunta, Matteo Incarbone, Davide Tosi, Luigi Santambrogio, Tommaso A. Dragani, Francesca Colombo

**Affiliations:** 1 Department of Predictive and Preventive Medicine, Milan, Italy; 2 Department of Experimental Oncology and Molecular Medicine, Fondazione IRCCS Istituto Nazionale dei Tumori, Milan, Italy; 3 Genomics of Renal Diseases and Hypertension Unit, IRCCS San Raffaele Scientific Institute, Milan, Italy; 4 School of Nephrology, Università Vita-Salute San Raffaele, Milan, Italy; 5 Department of Surgery, Ospedale San Giuseppe, Milan, Italy; 6 Fondazione IRCCS Cà Granda Ospedale Maggiore Policlinico, Milan, Italy

## Abstract

Many single nucleotide polymorphisms (SNPs) have been associated with lung cancer but lack confirmation and functional characterization. We retested the association of 56 candidate SNPs with lung adenocarcinoma risk and overall survival in a cohort of 823 Italian patients and 779 healthy controls, and assessed their function as expression quantitative trait loci (eQTLs). In the replication study, eight SNPs (rs401681, rs3019885, rs732765, rs2568494, rs16969968, rs6495309, rs11634351, and rs4105144) associated with lung adenocarcinoma risk and three (rs9557635, rs4105144, and rs735482) associated with survival. Five of these SNPs acted as *cis*-eQTLs, being associated with the transcription of *IREB2* (rs2568494, rs16969968, rs11634351, rs6495309), *PSMA4* (rs6495309) and *ERCC1* (rs735482), out of 10,821 genes analyzed in lung. For these three genes, we obtained experimental evidence of differential allelic expression in lung tissue, pointing to the existence of in-*cis* genomic variants that regulate their transcription. These results suggest that these SNPs exert their effects on cancer risk/outcome through the modulation of mRNA levels of their target genes.

The study of genetic factors modulating an individual’s predisposition to lung cancer is supported by strong epidemiological evidence obtained from various types of studies. Observational studies have consistently reported an increased risk of lung cancer in first-degree relatives of lung cancer patients^1^,^2^,^3^,^4^,^5^. Genome-wide association studies (GWAS) on population-based series identified three main susceptibility loci, at 5p15^6^,^7^,^8^, 6p21^6^, and 15q25. The locus at 15q25 harbors genes for three nicotinic acetylcholine receptor subunits (*CHRNA3, CHRNA5, and CHRNB4*) that have previously been associated with lung cancer risk and nicotine dependence^6^,^9^,^10^. Other GWAS found many single nucleotide polymorphisms (SNPs) that associated with lung cancer risk^11^,^12^,^13^,^14^,^15^,^16^. However, the results obtained in these studies have not generally been confirmed, even in a large consortium study^17^.

Some genetic variants associated with lung cancer risk have also been associated with prognosis. For instance, a polymorphism (rs6495309) in the promoter of *CHRNA3* gene has been reported to be associated with the overall survival of patients with early-stage non–small-cell lung cancer (NSCLC)^18^. Moreover, rs667282 in *CHRNA5* has recently been proposed as a modifier of prognosis in advanced NSCLC^19^. Several other SNPs, found using a GWAS approach, were proposed to be associated with prognosis or survival of lung cancer patients in different populations^20^,^21^,^22^,^23^. However, these studies did not identify the same candidate polymorphisms, which may be due (at least in part) to the wide genetic heterogeneity of the human population.

The GWAS cited here, which aimed to find SNPs associated with lung cancer risk or prognosis, identified mostly non-overlapping subsets of SNPs, hindering progress in lung cancer research. One limitation of these studies is that they investigated relatively small, often heterogeneous populations. Therefore, replication of these association studies in other case-control cohorts is warranted. Moreover, to go beyond statistical associations, these candidate SNPs must be investigated for their putative functional roles in lung cancer.

The functional characterization of candidate SNPs from GWAS is particularly challenging, since most of them map in non-coding regions^24^ and therefore do not exert direct effects on proteins, for example by introducing premature stop codons. These “regulatory SNPs” exert their effects by modifying non-transcribed regions of the genome (e.g. gene promoters, enhancers and silencers) where they alter transcription factor binding and chromatin states, as well as untranslated regions of RNA where they affect RNA splicing^25^,^26^. Therefore, these SNPs may modulate the expression of both near and distant genes; when these genes are involved in cancer-related pathways, the SNPs may affect the process of tumorigenesis. For instance, our finding that SNPs in the promoter of *CHRNA5* altered this gene’s expression levels in normal lung tissue^27^ suggested that modulation of transcriptional activity was, at least in part, responsible for the association of the 15q25 locus with lung cancer risk. These results shed light on a possible functional role of these polymorphisms in lung tumorigenesis.

In genetics, chromosomal loci that modulate gene expression–a quantitative trait–are called “expression quantitative trait loci” (eQTLs). eQTL analysis is a powerful genetic approach for testing if modulation of transcriptional activity is a mechanism by which SNPs affect a phenotype^28^,^29^. By crossing data on SNP genotypes with those on gene expression, this analysis enables the discovery of meaningful genotype-phenotype relationships^30^. Hence, the study of eQTLs in lung tissue may provide insight into mechanisms involved in lung tumorigenesis.

This study focused on 56 SNPs previously reported to be associated with lung cancer risk, survival, or factors predisposing to lung cancer, i.e., chronic obstructive pulmonary disease (COPD)^31^ and nicotine dependence^32^. First, we attempted to replicate their association with lung cancer risk and survival in a relatively large, uniform and well characterized series of lung cancer patients, with the same tumor histotype (adenocarcinoma) and similar durations of follow-up, and in sex-matched healthy controls. Additionally, we used eQTL analysis to look for the possible involvement of these candidate SNPs in the modulation of gene expression in non-involved lung tissue from lung adenocarcinoma patients, with the aim of identifying their mechanisms of action in lung cancer predisposition and progression.

## Results

### Patients’ clinical characteristics

This study investigated 823 lung adenocarcinoma patients and 779 sex-matched healthy controls, all from Italy (Table 1). The patients’ median age at surgery was 64 years, but there was a wide age range; the controls’ median age at recruitment was 61 years. The age difference between cases and controls was statistically significant (*P* = 3.9 × 10^−8^). Male sex predominated (~70%) among both cases and controls. Ever smokers were more frequent than never smokers in both cases (85%) and controls (98%), and the percentage of ever smokers in controls was significantly higher than in cases (*P* < 2.2 × 10^−16^). The higher number of ever smokers in the control group is due to the fact that most of them (621 out of 779) were recruited during a lung cancer screening program. About half of cases were at pathological stage I. The median follow-up period for patients who did not die during the study was 60 months, while at the 60-month follow-up 354 patients (44%) had died.

To identify clinical characteristics associated with overall survival, multivariate Cox analyses were performed (Table 1). Overall survival associated with sex, with men having shorter survival than women (HR = 1.6; 95% CI, 1.2–2.1; *P* = 1.2 × 10^−3^). Overall survival also associated with pathological stage, as expected, but it did not associate with age at surgery or smoking habit.

### Association between SNP genotypes and lung cancer risk or prognosis

A total of 64 SNPs, previously reported to associate with lung cancer risk or prognosis, were genotyped in cases and controls (Supplementary Table S1). Quality control filtering of raw data revealed that a 90% genotype call rate was not reached for 6 SNPs (rs1261411, rs2736100, rs6488007, rs6537296, rs503464, and rs55781567). Moreover, for two SNPs (rs3117582 and rs639739), the genotype data did not segregate into distinct clusters. Therefore, 8 SNPs were excluded while 56 SNPs were considered in the subsequent analyses.

To identify SNPs associated with lung cancer risk in our Italian series, we used logistic regression adjusting for sex, age, and smoking habit (Table 2). This replication analysis revealed statistically significant associations between lung adenocarcinoma risk and eight SNPs located on five chromosomes. In particular, we confirmed the association of rs401681 in an intronic region of *CLPTM1L* gene on chromosome 5, within the 5p15 susceptibility locus; the minor allele of rs401681 associated with a lower lung cancer risk (odds ratio, OR < 1). On chromosome 8, rs3019885 was confirmed to associate with lung cancer risk; with an OR > 1, the minor allele of this SNP is the risk allele. On chromosome 14, we found a weak association between a higher lung adenocarcinoma risk and the minor allele of rs732765, which had previously been associated with poorer survival in NSCLC (see Supplementary Table S1). We replicated the association with lung cancer risk for four SNPs in the nicotinic acetylcholine receptor locus on chromosome 15q25: rs2568494 (previously associated with COPD), rs16969968 (lung cancer risk and survival, and COPD), rs6495309 (lung cancer risk and survival), and rs11634351 (nicotine dependence). Three of these SNPs had an OR > 1, meaning that the minor alleles were risk alleles, while the fourth, rs6495309, had an OR < 1. Finally, we confirmed the association with lung cancer risk for rs4105144 upstream of *CYP2A6* gene on chromosome 19, whose minor allele showed a protective role (OR < 1). The strongest statistical association was found at rs2568494 (*P* = 6.00 × 10^−7^), located near the *IREB2* gene in the nicotinic acetylcholine receptor locus.

Next, to identify SNPs associated with overall survival in lung adenocarcinoma patients, we used a multivariable Cox proportional hazard model, adjusted for sex, age, smoking habit, and pathological stage (stage I versus stage > I). This analysis identified three SNPs on two chromosomes (Table 3). In particular, we confirmed the association with survival for rs9557635, an intronic polymorphism of *NALCN* gene. Moreover, we found that rs4105144, near *CYP2A6* gene on chromosome 19, was associated with survival as well as with risk (see Table 2). Finally, we replicated the association of rs735482 (in the 3’-UTR of *ERCC1* gene) with survival. The minor alleles of rs9557635 and rs4105144 were associated with poorer survival (HR > 1), whereas for rs735482 we observed the opposite effect (HR < 1).

The relationship between these three SNPs and survival was then visualized using Kaplan-Meier curves (Supplementary Fig. S1). Carriers of the minor allele of rs9557635 had worse probability of survival than homozygotes at the common allele (genotype GG, Supplementary Fig. S1A), in agreement with the HR of 1.17 (*P* = 0.035, see Table 3). For rs4105144, survival curves for heterozygotes and homozygotes at the common allele were almost undistinguishable (Supplementary Fig. S1B), whereas homozygous carriers of the minor allele (genotype TT) had worse survival than the others. For rs735482, heterozygotes had better survival than homozygotes at the major allele (Supplementary Fig. S1C), in agreement with the HR of 0.78 (*P* = 0.035; the small number (n = 16) of patients homozygous for the minor allele did not allow the effect of this genotype on survival to be assessed).

**Figure 1:**
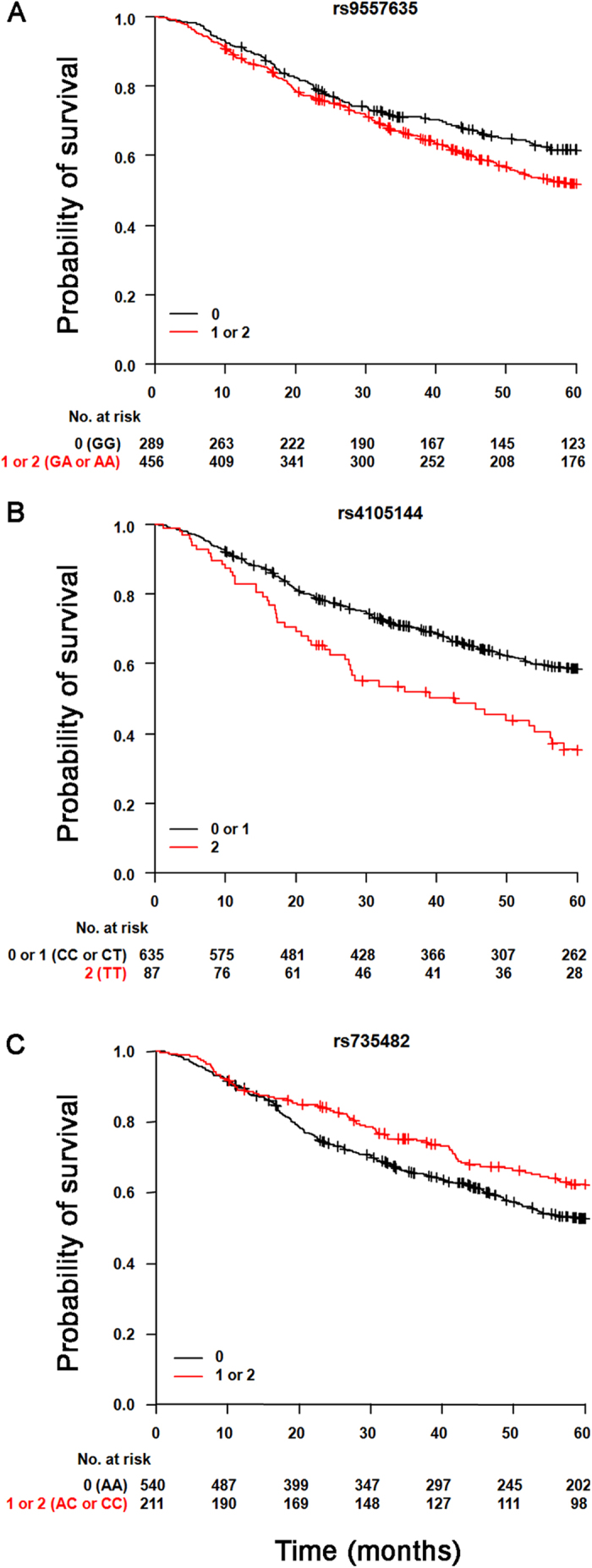
Kaplan-Meier survival curves for lung adenocarcinoma patients, according to the genotypes of three SNPs associated with overall survival. (**A**) For rs9557635, we tested a dominant model for the minor allele, and therefore compared carriers of the minor allele (red) with homozygotes for the common allele (black). (**B**) For rs4105144, we tested a recessive model for the minor allele, and so compared homozygotes for the minor allele (red) with carriers for the common allele (black). (**C**) For rs735482, we tested a dominant model for the minor allele, and therefore compared carriers of the minor allele (red) with homozygotes for the common allele (black). Crosses denote censored samples. Below the figures are reported the number of patients at risk at the specified times of follow-up.

On the basis of this analysis, we repeated Cox’s analysis after having grouped genotypes according to a dominant or recessive model (Fig. 1). For rs9557635, we compared individuals carrying at least one copy of the minor allele with homozygotes for the common allele, as in a dominant model, and found that minor allele carriers had a higher risk of death (HR = 1.31, *P* = 0.018) than homozygotes for the common allele (Fig. 1A). For rs4105144 (Fig. 1B), we compared homozygotes at the minor allele to carriers of the common allele, and observed a much stronger statistical association with survival than when we assessed the dosage effect of the minor allele (HR = 1.90, *P* = 4.6 × 10^−5^ vs. *P* = 0.025 in Table 3). This result suggests that, for rs4105144 or a functional variation in tight linkage disequilibrium with it, the minor allele has a recessive effect on overall survival of lung adenocarcinoma patients. Finally, for rs735482 (Fig. 1C), we compared individuals carrying at least one copy of the minor allele with homozygotes for the common allele, and found a significantly better survival (HR = 0.73, *P* = 0.014) for carriers of the minor allele; this result suggests a possible dominant protective effect of the minor allele on survival of lung adenocarcinoma patients.

Collectively, this analysis of 56 candidate SNPs previously reported to be associated with lung cancer confirmed, in a relatively large population homogeneous for both histotype and ethnicity, the association for ten SNPs: seven with lung adenocarcinoma risk, two with survival, and one with both risk and survival. Because the original studies that discovered these associations were carried out in populations different from the one studied here, this replication study suggests that these ten SNPs have broad importance in lung adenocarcinoma, irrespective of ethnic group.

### Some lung cancer-related SNPs are putative eQTLs

Because most candidate SNPs identified with GWAS map to non-coding regions of the genome, it is plausible that they exert their cellular effects via the modulation of gene expression. To test this possibility for the ten confirmed lung cancer-related SNPs, we carried out an expression quantitative trait loci (eQTL) analysis in the non-involved lung parenchyma of lung adenocarcinoma patients. In particular, we looked for correlations between the genotypes of these SNPs and the expression levels of 10,821 genes in 232 cases. This analysis identified six *cis*-eQTLs, involving five SNPs associated with the expression of three genes (Table 4). There are more *cis*-eQTLs than SNPs because one SNP (rs6495309) is associated with expression levels of two transcripts (*IREB2* and *PSMA4*). In detail, *IREB2* on chromosome 15 (at ∼78 Mbp, on Assembly GRCh38) was found to be regulated by four SNPs (rs2568494, rs16969968, rs6495309 and rs11634351); in the same locus, rs6495309 is associated also with *PSMA4* mRNA levels. Additionally, *ERCC1* on chromosome 19 (at ∼45 Mbp) associated with one SNP (rs735482). Two eQTL SNPs are located in the gene they modulate: rs2568494 maps in an intronic region of *IREB2* gene and rs735482 maps in the 3′-UTR of *ERCC1* gene. This latter SNP also maps in the coding sequence of *CD3EAP*, a gene that overlaps with *ERCC1* but is transcribed in the opposite direction. No *trans*-eQTLs were found. Altogether, this eQTL analysis provides statistical evidence for the in-*cis* regulation of three genes by six genetic elements associated with lung cancer risk or survival.

We repeated the eQTL analysis considering all 56 SNPs included in the present study (Supplementary Table S2). This analysis found 10 *cis*-eQTLs, including four of those described above: the associations of rs11634351 and rs6495309 with IREB2 were lost, probably as a consequence of a power reduction in this wider analysis. An additional locus on chromosome 15 (at ~43 Mbp, on Assembly GRCh38) was identified, where rs504417 associated with the expression of *ADAL* gene. Also rs578776, in the nicotinic acetylcholine receptor locus, was strongly associated with *IREB2* expression. Of note, this same SNP, together with rs6495309, associated with mRNA levels of *PSMA4*, another gene in the chromosome 15q25 locus. On chromosome 19, this wider eQTL analysis identified two additional SNPs (rs1005165 and rs967591) associated with *ERCC1* levels; one of them (rs967591) also associated with *VASP* gene expression. No *trans*-eQTLs were found.

### Genetic elements located in cis of ERCC1, IREB2, and PSMA4 regulate expression in an allelic-specific manner

**Figure 2:**
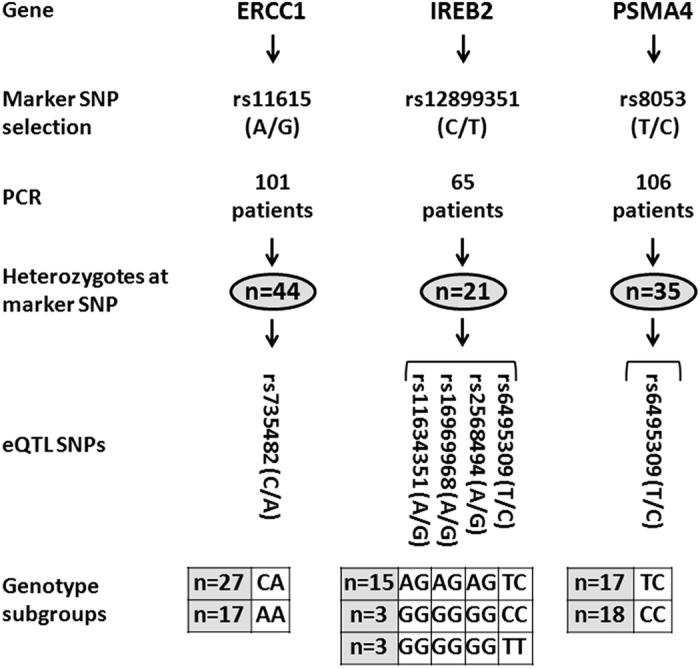
Workflow for DAE assay. For each putative target gene of eQTL SNPs, we chose a marker SNP in the transcribed region (shown are minor/common alleles). Heterozygous patients were selected by pyrosequencing genomic DNA with SNP-specific PCR primers (Supplementary Table S3). Heterozygotes were then subgrouped according to their genotypes at the eQTL SNPs to create the comparison groups for the DAE assay.

To experimentally test the results of the eQTL analysis, we examined if the two putatively regulated genes exhibited differential allelic expression (DAE). We selected lung adenocarcinoma patients who were heterozygous at a marker SNP in each gene, and subgrouped them on the basis of their genotypes at the eQTL SNPs (Fig. 2). cDNA from the patients’ non-involved lung tissue was PCR-amplified in the region spanning the marker SNP, and allelic expression was determined by pyrosequencing the PCR products. Since the marker SNPs were in loose linkage disequilibrium with the eQTL SNPs (data not shown), we stratified the patients into groups according to whether they were heterozygous or homozygous for the eQTL SNPs before assessing DAE.

**Figure 3:**
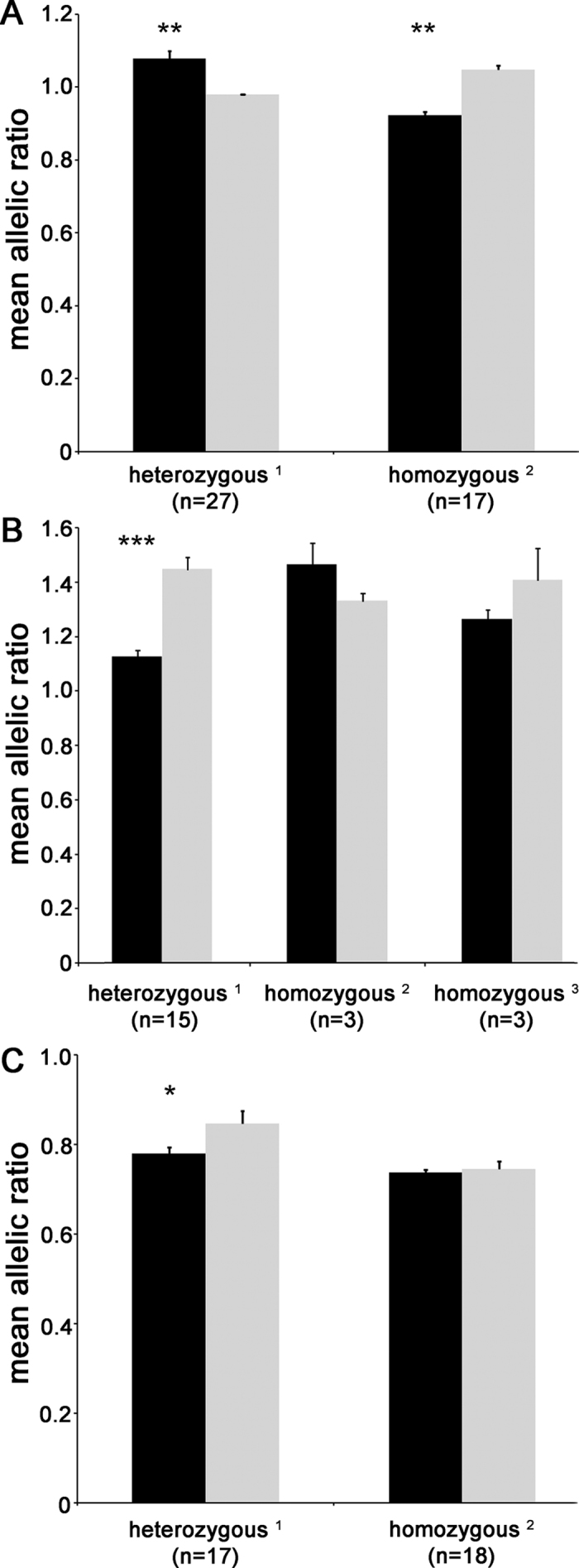
Differential allelic expression in *ERCC1, IREB2*, and *PSMA4* supports the existence of *cis*-acting genetic regulations. Allelic ratios (minor/common allele) for marker SNPs in cDNA (black bars) and genomic DNA (gray bars) from patients heterozygous for (**A**) rs11615 in *ERCC1* gene, (**B**) rs12899351 in *IREB2* gene, and (**C**) rs8053 in *PSMA4* gene. Marker SNPs map in gene transcribed regions and, thus, can be genotyped in both cDNA and genomic DNA. Data are means and SE. Patients are subgrouped according to their genotypes at the eQTL SNPs for each gene. In particular, the heterozygous^1^ subgroup consisted of cases that were heterozygous at all eQTL SNPs, while the homozygous^2^ subgroup had cases that were homozygous for the common allele at all eQTL SNPs, and the third subgroup, homozygous^3^, formed for *IREB2* gene only, consisted of cases that were homozygous for the common allele at three eQTL SNPs (rs2568494, rs16969968, and rs11634351) and homozygous for the minor allele at the fourth eQTL SNP (rs6495309) (see Fig. 2). **P < 0.001; ***P < 0.0001 (Wilcoxon’s signed rank test on paired log_10_-transformed allelic ratios).

The ratios of the minor to common alleles of marker SNPs in cDNA were compared to those obtained in genomic DNA, in the genotype subgroups for each gene (Fig. 3). For *ERCC1* gene (Fig. 3A), the ratios for cDNA were significantly different from those for genomic DNA, indicating DAE, in both subgroups of patients. In particular, in patients heterozygous for rs735482 eQTL SNP there was more expression of the minor than common allele, while in homozygous patients there was less expression of the minor allele (*P* = 4.0 × 10^−4^ and *P* = 8.4 × 10^−4^, respectively; Wilcoxon’s test on paired log_10_-transformed allelic ratios). For *IREB2* (Fig. 3B), there was evidence of DAE in patients who were heterozygous at all four eQTL SNPs (rs2568494, rs16969968, rs6495309 and rs11634351), with lower expression of the minor than common allele (*P* = 6.1 × 10^−5^), but not in the two groups of patients who were homozygous for these SNPs. Similar results were obtained for *PSMA4 (P* = 0.015, Fig. 3C).

## Discussion

This study focused on 56 SNPs previously reported to be associated with lung cancer in different populations, and comprised both a replication effort and an eQTL analysis. In the replication study, eight SNPs were found to associate with the risk of lung adenocarcinoma in our Italian series. Additionally, three SNPs were found to associate with overall survival in this cohort. Altogether, the replication study confirmed an association with lung cancer for 10 SNPs, as one SNP associated with both risk and survival. In the eQTL analysis, the genotypes of five of these ten SNPs were found to associate with the expression levels of three genes on the same chromosomes, indicating the presence of *cis*-eQTLs, in 232 samples of non-involved lung tissue from lung adenocarcinoma patients. For these genes (*IREB2, PSMA4* and *ERCC1*), we found evidence of DAE, suggesting that the SNPs exert their effects on lung tissue by modulating gene expression.

In the replication study, eight SNPs associated with lung cancer risk in our case-control cohort. Because the cohort had significantly fewer ever smokers among cases (85%) than controls (98%), these SNPs are likely to be associated directly with lung cancer risk rather than indirectly through an association with smoking behavior.

Among the SNPs that associated with lung cancer risk, four (rs2568494, rs16969968, rs6495309, and rs11634351) were in the nicotinic acetylcholine receptor locus on chromosome 15. rs2568494, previously reported to be associated with COPD^33^,^34^, is an A/G variant in *IREB2*. This SNP is included in the *IREB2* AAAT haplotype (rs2568494, rs2656069, rs10851906, rs13180) that has recently been associated with lung cancer risk (OR  =  1.5)^35^. Our data confirm the association of the A allele of rs2568494 with increased lung cancer risk. The second SNP, rs16969968, is a G/A variant in the coding sequence of *CHRNA5*. This SNP associated with smoking-related traits such as nicotine dependence^36^, COPD^37^, smoking cessation treatment benefit^38^, and smoking quantity^10^. Our study confirmed the association of its minor allele (A) with increasing lung cancer risk^9^,^39^. The third SNP, rs6495309, is an intergenic C/T polymorphism mapping between *CHRNA3* and *CHRNB4* genes; it previously was associated with lung cancer risk^40^, survival^18^ and COPD^37^. Our study confirms the association of its minor allele (T) with decreasing lung cancer risk. Finally, rs11634351 is a G/A intron variant in *CHRNB4* gene that has never been reported to be associated with lung cancer risk, but was found to modulate nicotine dependence and smoking-related phenotypes^41^. Our finding of a significant association of the A allele of rs11634351 with increased lung cancer risk may be due to its linkage disequilibrium with another variant with a functional role or may indicate the genetic complexity of the locus in modulating lung cancer risk.

Also associating with lung cancer risk was rs401681, a C/T intronic variant in *CLPTM1L* gene on chromosome 5p15.33; the T allele of rs401681 was previously found to associate with a lower risk of lung cancer in two meta-analyses^42^,^43^, and our results confirm these findings. On chromosome 8, we confirmed our previously observed association of the minor allele (G) of rs3019885 with increasing lung cancer risk^15^. On chromosome 14, a significant association with lung cancer risk was found for the minor allele (G) of rs732765, which, in a Korean study^22^, was associated with poorer survival of NSCLC patients.

Finally, our study confirmed an association with lung cancer risk for the minor (T) allele of rs4105144^44^. This SNP, which also associates with smoking behavior^45^, is a T/C variant upstream of *CYP2A6*, which encodes cytochrome P450 2A6^46^. This cytochrome is the main nicotine-metabolizing enzyme, and it is also involved in the bioactivation of carcinogens present in tobacco smoke^47^. *CYP2A6* has several allelic variants that affect the rate of nicotine metabolism^48^. Because rs4105144 is in linkage disequilibrium with the slow metabolizing alleles, the association of the minor allele with lower lung cancer risk can be explained by a protective action against carcinogen activation.

In the analysis of overall survival, three SNPs were identified, including rs4105144 which we also confirmed to be associated with lung cancer risk. The association of this SNP with lung cancer survival is a novel finding, and our observation that homozygous carriers of the T allele had poorer survival than carriers of the common allele (*P* = 4.6 × 10^−5^) suggests a potential functional involvement of the recessive minor allele. This result contrasts with the protective role of the T allele in lung cancer risk; however, it has already been reported that an increasing number of T alleles associates with poorer survival in gastric cancer patients treated with adjuvant chemotherapy^49^. The mechanism underlying such association is not known but may involve poor biotransformation of chemotherapeutic agents or endogenous compounds such as retinoic acids and steroids^50^, which, in turn, might influence survival of lung adenocarcinoma patients.

Our study confirmed the association with lung cancer survival of rs9557635 (in *NALCN* gene) and found that its minor allele (A) associated with poorer prognosis, as previously reported^22^. Finally, for rs735482 (mapping in overlapping genes *CD3EAP* and *ERCC1* on chromosome 19), we observed a significant association of the minor allele (C) with better prognosis, differently from studies that associated the same C allele with poorer prognosis^22^,^51^. For both these SNPs we observed a dominance effect of the minor allele on survival of lung adenocarcinoma patients.

Overall, our study confirmed few of the previously reported associations of the 56 SNPs with lung cancer risk and survival. This failure is probably due to the fact that most associations were found in heterogeneous populations, characterized by patients with different lung cancer histotypes. Instead, we studied only a single lung cancer histotype (adenocarcinoma), limited to surgically treated patients and to a single country (Italy). An additional explanation is possible confounding effects of somatic mutations on prognosis. Indeed, some somatic mutations, in particular *KRAS* mutations, associated with poorer survival of NSCLC patients^52^. However, other mutations (in *EGFR, TP53, PIK3CA*, and also in *KRAS*) did not associate with survival in different populations^53^,^54^,^55^,^56^. Therefore, we hypothesize that germline polymorphisms and somatic mutations interact and cooperate in modulating lung cancer patients’ survival, by acting on or interfering with the same molecular pathways. This possibility should be investigated further.

We previously hypothesized^57^ that individual predisposition to lung cancer is already detectable in non-involved lung tissue and that polymorphisms which associate with lung cancer risk or prognosis act by modulating gene expression levels in lung tissue. Therefore, we assessed the confirmed ten lung cancer-related SNPs for possible roles as eQTLs, and found a significant (FDR < 0.05) effect of five SNPs in the modulation of mRNA levels of three genes on the same chromosomes, hence functioning as *cis*-eQTLs. In particular, four risk-associated SNPs in the nicotinic acetylcholine receptor locus on chromosome 15 (rs2568494 in *IREB2;* rs16969968 in *CHRNA5*; rs6495309 in *CHRNA3*/*CHRNB4*; and rs11634351 in *CHRNB4*) and one survival-associated SNP (rs735482 in *CD3EAP* and *ERCC1* on chromosome 19), were found to associate with mRNA levels of *IREB2* (rs2568494, rs16969968, rs6495309 and rs11634351), of *PSMA4* (rs6495309), and of *ERCC1* (rs735482) genes. We did not confirm previous reports of nicotinic acetylcholine receptor locus polymorphisms modulating *CHRNA5* levels^27^,^58^,^59^. This unexpected result may be due to the existence, in non-involved lung tissue, of *CHRNA5* transcript variants that were not detected by the gene expression microarray we used. Indeed, our previous findings of polymorphisms modulating *CHRNA5* levels were obtained using qPCR^27^,^58^.

To test whether the statistical associations between the five eQTL SNPs and expression levels of the three target genes are attributable to variations in *cis*-regulatory elements mapping close to these eQTL SNPs, we examined the possibility of differential allelic expression (DAE) of the target genes. Indeed, the allelic-specific transcription of a gene indicates that genetic elements in *cis* to it (e.g. in its promoter or untranslated regions of its RNA) modulate its expression. Of note, we found the existence of *cis* regulatory variations for the three genes we tested, i.e. *IREB2, PSMA4*, and *ERCC1*. For *IREB2* and *PSMA4* genes, we detected statistically significant DAE in patients heterozygous at all the eQTL SNPs, whereas no significant association was found in patients homozygous for the same SNPs. Therefore, these results suggest that the eQTL SNPs are in linkage disequilibrium with *cis*-acting variations, yet unidentified, modulating *IREB2* expression level. At the *ERCC1* gene, instead, statistically significant DAE was detected in patients both heterozygous and homozygous at the SNP modulating its expression levels. These results might be due to a complex pattern of linkage disequilibrium in the analyzed locus and to the putative existence of more than one functional variation affecting *ERCC1* expression. For all target genes herein examined, their functional *cis*-acting elements have not yet been identified.

Our findings of *IREB2* DAE in non-involved lung tissue confirm previous results obtained in lung tumor tissue. Fehringer *et al*.^60^, who studied two series of lung tumor tissue from NSCLC patients, reported association of rs16969968 with *IREB2* levels in one of the two series. The direction of effects (the sign of the beta parameter in Table 4) of the minor alleles on *IREB2* mRNA levels in our study are the same as in that study. Together, these results provide convincing evidence that SNPs in the nicotinic acetylcholine receptor locus on chromosome 15 modulate expression levels of *IREB2* and, thus, implicate *IREB2* expression levels in the individual risk for lung cancer. Because *IREB2* encodes an RNA binding protein involved in iron metabolism^61^, it has been suggested that high levels of iron in the lung are a predisposing factor for high oxidative stress, high inflammation and, therefore, lung cancer^60^,^62^. Accordingly, our results suggest that lower expression of *IREB2* gene, in the presence of the minor allele of rs2568494 (beta parameter, −0.210; Table 2), confers a higher risk of lung cancer (OR = 1.437).

As far as *ERCC1* is concerned, DAE was already reported at both mRNA and protein levels in prostate cancer at rs11615, with the C allele expressed at lower levels than the T allele^63^. Additionally, a better response to chemotherapy was reported for lung cancer patients carrying the C allele^64^. *ERCC1* is a key player of the nucleotide excision repair (NER) system, which is activated by chemotherapeutic agents that induce DNA damage in cancer cells to kill them^65^. Therefore, the NER pathway and, in particular, *ERCC1* activation are frequently responsible for chemoresistance. Our eQTL and survival results indicate that lower *ERCC1* expression in non-involved lung tissue of adenocarcinoma patients, in presence of the C allele, was associated with better outcome. These results can be explained by a lower activity of the NER pathway in patients with lower *ERCC1* expression. Although we do not have data about chemotherapy treatment in our patient series, we are confident that patients with advanced stage lung adenocarcinoma underwent chemotherapy since clinical management of these patients usually includes this treatment^66^,^67^. Therefore, we can suppose that the better survival observed in patients with allele C of *ERCC1* rs11615 polymorphism is, at least in part, due to the reduced functionality of *ERCC1* pathway.

In conclusion, our study highlights the relevance of deepening the function of the many loci found associated with human complex traits by GWAS, and points to the role of some of these loci as eQTLs. Our results support the hypothesis that some polymorphisms associated with lung cancer risk or prognosis influence tumor development and progression through the modulation of expression levels of target genes. Functional studies are needed to reach a more complete picture of the molecular mechanisms mediated by such SNP-specific alterations in gene expression, underpinning cancer pathogenesis.

## Methods

### Population series and biological material

The population series investigated in this study comprised 823 lung adenocarcinoma patients (cases) and 779 healthy controls. Cases were patients who had undergone lobectomy at one of three hospitals in the area around Milan, Italy (Fondazione IRCCS Istituto Nazionale dei Tumori, San Giuseppe Hospital, Fondazione IRCCS Cà Granda Ospedale Maggiore Policlinico). Samples of peripheral blood and non-involved (apparently normal) lung parenchyma, recovered during lobectomy, had been taken for research purposes. This group included 232 patients for whom lung transcriptome data had already been obtained in a previous study^57^. Control subjects were recruited among blood donors (n = 158) and participants in a lung cancer screening program (n = 621), and were matched to cases by sex. Control subjects donated a sample of peripheral blood.

After collection, tissue and blood samples were used to extract RNA (from tissue only) and genomic DNA (tissue and blood). These materials were stored in the biobank of Fondazione IRCCS Istituto Nazionale dei Tumori. Methods for the collection of samples and associated clinical data have already been reported^15^,^20^,^57^. The Committees for Ethics of the institutes involved in recruitment (Fondazione IRCCS Istituto Nazionale dei Tumori, Milan, Italy, Ospedale San Giuseppe, Milan, Italy, Fondazione IRCCS Cà Granda Ospedale Maggiore Policlinico, Milan, Italy) approved the protocol for collecting samples and clinical data. Cases and controls provided written informed consent for the use of their biological material and data for research purposes. All methods were performed in accordance with the relevant guidelines and regulations.

### SNP selection and genotyping

We selected 64 SNPs to genotype based on our previous GWAS results and on GWAS, meta-analyses, and follow-up characterization studies that had been carried out from 2008 to December 31, 2013 (Supplementary Table S1).

For genotyping, we used commercially available or custom TaqMan SNP Genotyping Assays (Supplementary Table S1). SNPs were genotyped using the TaqMan Open Array Genotyping System (Thermo Fisher Scientific, Waltham, USA). DNA samples were loaded at 50 ng/mL and amplified according to the manufacturer’s instructions. For analysis of the genotypes, we used autocalling methods, as implemented in the TaqMan Genotyper software version 1.3. A genotype call rate ≥0.90 was selected as the reliability threshold; this means that SNPs for which it was impossible to determine the genotype for more than 10% of samples were eliminated from consideration.

### Detection of eQTLs

To identify which, if any, lung cancer-related SNPs influence gene expression in normal lung tissue (i.e. function as eQTLs), we took advantage of transcriptome data that we had previously generated from the non-involved lung parenchyma of 284 Italian lung adenocarcinoma patients (all smokers)^57^. In that study, gene expression profiling had been done on HumanHT-12 v4 Expression BeadChip microarrays (Illumina) according to a discovery–validation design. Here, the two datasets of 206 samples (discovery series) and 78 samples (validation series) were combined using the ComBat adjustment method^68^ implemented in the *sva* R package^69^. Probes that were not annotated and those with a detection *P* value < 0.01 in fewer than 10% of samples were filtered out. When multiple probes mapped to the same transcript, we included only the one with the highest detection rate, defined as the percentage of samples in which the probe had detection *P* values < 0.01. These expression data (for 10,821 genes) have already been deposited in the Gene Expression Omnibus database (GEO, http://www.ncbi.nlm.nih.gov/geo/) with accession number GSE71181.

For this study, we excluded from analysis 52 of these patients for whom genomic DNA was not available for genotyping. Thus, the eQTL analysis was done with 232 cases. First, the coordinates of the genotyped SNPs and the 10,821 genes with expression data were updated to the human genome assembly GRCh38 (Ensembl release 78) using the biomaRt R package^70^. Then, using MatrixEQTL R package^71^, we examined the correlations between the genotype at each SNP and the expression level of each gene, following the standard additive linear regression model which assumes that genotype has an additive effect on gene expression. For this analysis, genotypes were expressed as integers (0, 1, or 2) according to the number of minor alleles at each SNP, and patient’s sex and age at surgery were used as covariates. The statistical significance of the detected eQTLs was first determined by the software using the *t* statistic, and then adjusted for multiple testing using the Benjamini-Hochberg procedure to obtain the false discovery rate (FDR) of each eQTL. The threshold significance level was set as an FDR < 0.05. SNPs whose genotypes were significantly associated with the expression levels of genes mapping within 1 Mbp of genomic distance were defined as *cis*-eQTLs, whereas all others were regarded as *trans*-eQTLs.

### Differential allelic expression assay

The genes found to be subject to eQTL modulation were tested for the possibility of differential allelic expression (DAE). For the DAE assay, we selected a single marker SNP located within the transcribed region of each gene of interest, and we compared the allelic frequencies of the marker SNPs between cDNA and genomic DNA from the non-involved lung tissue of lung adenocarcinoma patients who were heterozygous at that SNP (Fig. 2). Marker SNPs had to have an arbitrarily selected minor allele frequency (MAF) >0.25 in the European population and be in a sequence context that permitted the design of a pyrosequencing assay. To identify heterozygous individuals at the marker SNPs, we genotyped subsets of patients chosen on the basis of their eQTL SNPs’ genotypes. SNP genotyping was carried out on a PyroMark Q96 ID system running PyroMark Q96 ID Software (Qiagen). Primer sequences for all PCR steps and pyrosequencing are reported in Supplementary Table S3.

Next, for each gene, we grouped the heterozygous cases (at the marker SNP) into subgroups depending on their genotypes at the eQTL SNPs. For example, one subgroup consisted of cases that were heterozygous at all eQTL SNPs for the particular gene. The formation of these subgroups was dictated by the genotype combinations that were observed, which depends on the allele frequencies and linkage disequilibrium patterns of the eQTL SNPs.

Then, for all the cases chosen for analysis, we synthesized cDNA from 1 μg of RNA extracted from non-involved lung tissue using the Transcriptor First Strand cDNA Synthesis Kit (Roche, Basel, Switzerland). To eliminate possible genomic DNA contamination, the cDNA (40 ng) was amplified with cDNA-specific primers (first amplification, Supplementary Table S3). Finally, this PCR product (4% of the reaction volume) and a sample of genomic DNA (40 ng) from the same patient were PCR-amplified using SNP-specific primers, in different tubes at the same time in parallel (second amplification, Supplementary Table S3). These PCR products were pyrosequenced on a PyroMark Q96 ID system running PyroMark Q96 ID Software (Qiagen). The proportions of individual alleles for each SNP were obtained from peak heights using the Pyro Mark MD software package (Qiagen), and allelic ratios (minor/common allele) for cDNA and genomic DNA were calculated for each case.

### Statistical analyses

Clinical characteristics were compared between cases and controls using Kruskal-Wallis test for the quantitative variable age and chi-square test for categorical variables. The associations between clinical characteristics and overall survival in cases were assessed using multivariate Cox proportional hazard test, using EZR in R Commander software^72^.

SNP genotypes were compared between cases and controls to identify genetic variants associated with lung cancer risk. For this purpose, the genotype at each SNP was coded with a numerical value (0, 1, or 2) according to the number of minor alleles present in order to represent the additive effects of the minor allele on the risk of lung cancer. The statistical analysis was done in a logistic model with sex, age, and smoker status as covariates, using PLINK software^73^. Odds ratios were calculated using the allele counting method, which assumes an additive effect of a variant, i.e., an OR > 1 means that the risk of lung adenocarcinoma increases with each copy of the minor allele. Since this was a validation study, we used a value of *P* < 0.05 to determine statistical significance.

To identify SNPs associated with overall survival of lung adenocarcinoma patients, we used multivariable Cox proportional hazard models. Again, the genotype at each SNP was coded with a numerical value according to the number of minor alleles present to represent the dosage effect of the minor allele on the risk of dying. In separate analyses, we compared homozygotes for the minor allele (code 2) to carriers of the common allele (code 0 or 1). In all analyses, the data were adjusted for sex, age, pathological stage (I versus > I), and smoker status. A value of *P* < 0.05 indicated statistical significance. To visualize the relation between carrier status of genotypes and overall survival, we used the Kaplan-Meier method.

For analysis of DAE, allelic ratios were aggregated into means and standard errors (SE), for cDNA and genomic DNA, by genotype subgroup. The data were also log_10_-transformed for statistical assessment using Wilcoxon’s signed-rank test for paired samples in R package; *P* < 0.05 indicated statistical significance.

## Additional Information

**How to cite this article**: Pintarelli, G. *et al*. Genetic susceptibility variants for lung cancer: replication study and assessment as expression quantitative trait loci. *Sci. Rep.* **7**, 42185; doi: 10.1038/srep42185 (2017).

**Publisher's note:** Springer Nature remains neutral with regard to jurisdictional claims in published maps and institutional affiliations.

## Supplementary Material

Supplementary Tables and Figure

## Figures and Tables

**Table 1:** Clinical characteristics of Italian lung adenocarcinoma patients (cases) and healthy controls.

Characteristic	Cases (n = 823)	Controls (n = 779)	Case-control comparison *P*^a^	Association with overall survival^b^	
				HR (95% CI)	*P*
Age, years, median (range)	64 (29–84)	61 (32–78)	3.9 × 10^−8^	1.0 (1.0–1.0)	0.06
Sex, n			0.33		
*Female*	254	223		1.0	
*Male*	569	556		1.6 (1.2–2.1)	1.2 × 10^−3^
Smoking habit, n			<2.2 × 10^−16^		
*Never*	116	15		1.0	
*Ever*	689	758		1.1 (0.8–1.6)	0.58
*Missing data*	18	6			
Pathological stage, n^c^			NA		
*I*	410	NA		1.0	
*II*	137	NA		2.9 (2.2–3.9)	4.3 × 10^−12^
*III or IV*	247	NA		4.5 (3.5–5.8)	<2.2 × 10^−16^
*Missing data*	29	0			
Cases with follow-up data, n	800	NA	NA		
Median (range) follow-up of patients alive, months	60 (10–60)	NA	NA		
Dead at the 60-month follow-up, n	354	NA	NA		

^a^Comparison between cases and controls; Kruskal-Wallis test for the quantitative variable age, chi-square test for categorical variables.

^b^HR, hazard ratio; CI, confidence interval; Cox multivariate test on cases only.

^c^Pathological stage, assessed after surgery.

NA, not applicable.

**Table 2:** SNPs associated with lung adenocarcinoma risk in logistic analysis (*P* < 0.05) in the Italian series of lung adenocarcinoma patients (n = 823) and healthy controls (n = 779)^a^.

SNP	Chr.	Position (bp)^b^	Closest gene	Minor allele	OR (95% CI)	*P*
rs401681	5	1,321,972	*CLPTM1L*	T	0.80 (0.69–0.93)	2.7 × 10^−3^
rs3019885	8	117,013,406	*SLC30A8*	G	1.21 (1.04–1.41)	0.012
rs732765	14	74,899,026	*DLST*	G	1.22 (1.02–1.47)	0.032
rs2568494	15	78,448,622	*IREB2*	A	1.44 (1.25–1.67)	6.00 × 10^−7^
rs16969968	15	78,590,583	*CHRNA5*	A	1.34 (1.16–1.55)	6.55 × 10^−5^
rs6495309	15	78,622,903	*CHRNB4*-*CHRNA3*	T	0.83 (0.69–1.00)	0.049
rs11634351	15	78,652,376	*CHRNB4*	A	1.27 (1.10–1.47)	1.4 × 10^−3^
rs4105144	19	40,852,719	*CYP2A6*	T	0.74 (0.63–0.87)	2.6 × 10^−4^

^a^Logistic analysis carried out in PLINK, adjusted for sex, age, and smoking habit, and based on additive effects of SNPs, i.e., an OR > 1 means that the risk of lung adenocarcinoma increases with the number of minor alleles.

^b^Based on Assembly GRCh38.p5.

**Table 3:** SNPs associated with overall survival of lung adenocarcinoma patients (n = 823) in a multivariable Cox proportional hazard model at P < 0.05.

SNP	Chr.	Position (bp)^a^	Closest genes	Minor allele	HR (95% CI)^b^	*P*
rs9557635	13	101,398,739	*NALCN*	A	1.17 (1.01–1.36)	0.035
rs4105144	19	40,852,719	*CYP2A6*	T	1.21 (1.02–1.44)	0.025
rs735482	19	45,408,744	*CD3EAP; ERCC1*	C	0.78 (0.62–0.98)	0.035

^a^Map position based on genome assembly GRCh38.p5.

^b^Hazard ratio for additive effects (allele dosage) of the minor allele (genotypes were coded with a numerical value, i.e., 0, 1, or 2, according to the number of minor alleles present) on the risk of dying; Cox’s analysis was adjusted by sex, age, pathological stage (I versus > I), and smoking habit; follow-up until 60 months.

**Table 4:** *cis*-eQTLs and their same-chromosome target genes identified in non-involved lung tissue from 232 lung adenocarcinoma patients (analysis limited to 10 confirmed SNPs. eQTLs clustered into two main loci.

eQTL	SNP	Chr.	Position (bp)^a^	Target gene	Nominal *P*	FDR	Beta^b^
1	rs2568494	15	78,448,622	*IREB2*	1.35 × 10^−6^	0.00018	−0.21
2	rs16969968	15	78,590,583	*IREB2*	5.70 × 10^−6^	0.00035	−0.20
3	rs11634351	15	78,652,376	*IREB2*	0.00158	0.035	−0.15
4	rs6495309	15	78,622,903	*IREB2*	0.000218	0.0098	0.21
5	rs6495309	15	78,622,903	*PSMA4*	0.00100	0.027	0.092
6	rs735482	19	45,408,744	*ERCC1*	0.000711	0.029	−0.12

^a^Map position based on genome assembly GRCh38.p5.

^b^Beta values are from the additive linear regression model (in MatrixEQTL R package), where genotype (expressed as the minor allele count) is assumed to have an additive effect on gene expression. Patients’ sex and age at surgery were used as covariates. A positive value indicates that the expression of the target gene increases with an increase in minor allele count.
