# Supplementary material for: Genetic susceptibility variants for lung cancer: replication study and assessment as expression quantitative trait loci
Source: Sci Rep. 2017 Feb 9;7:42185. doi: 10.1038/srep42185 (PMC5299838; doi:10.1038/srep42185)
Supplement: Supplementary Tables and Figure [file srep42185-s1.pdf]

Genetic susceptibility variants for lung cancer: replication study and assessment as expression quantitative trait loci

Giulia Pintarelli, Chiara Elisabetta Cotroneo, Sara Noci, Matteo Dugo, Antonella Galvan, Simona Delli Carpini, Lorena Citterio, Paolo Manunta, Matteo Incarbone, Davide Tosi, Luigi Santambrogio, Tommaso A. Dragani, Francesca Colombo

Supplementary Table S1. Properties of 64 SNPs associated with risk or clinically relevant parameters in lung cancer, according to published studies, and genotyping data from 823 lung adenocarcinoma patients and 779 healthy controls. Quality control on genotype data excluded 8 SNPs from further analysis

Chr. [1]	Map position (bp) [1]	SNP	Closest gene	Original publication			TaqMan assay [2]	Minor allele [3]	Common allele [3]	MAF [3]
				Role	p-value	Reference				
1	4329202	rs639739	NA	risk	0.0124	[1]1	C____587595_10	NA	NA	NA
1	56552066	rs1261411	PPAP2B	risk	0.0114	[1]1	C___8327128_10	NA	NA	NA
2	36255059	rs2699164	CRIM1	survival	1.06×10^{-5}	[2]2	C__16281545_20	C	T	0.117
2	137384594	rs13405020	THSD7B	survival	6.52×10^{-6}	[2]2	C__30984975_10	C	G	0.163
2	168214838	rs4438452	STK39	survival	9.97×10^{-6}	[3]3	C__11637282_10	T	C	0.190
2	210704998	rs2371030	CPS1	survival	2.58×10^{-6}	[2]2	C__16214889_10	G	A	0.361
3	14393563	rs721377	SLC6A6	risk	0.0126	[1]1	C____913863_10	A	G	0.066
3	40925416	rs7629386	NA	survival	3.63×10^{-5}	[4]4	C___1468486_10	T	C	0.311
3	190632672	rs7626795	IL1RAP	risk	7.80×10^{-6}	[5]5	C__29825255_20	G	A	0.162
4	30772461	rs10517215	PCDH7	survival	2.45×10^{-5}	[3]3	C__30566442_20	A	C	0.130
4	40337557	rs6819385	CHRNA9	risk	0.002	[6]6	C__25993305_10	G	A	0.454
4	65100590	rs1877116	EPHA5	risk	0.0273	[7]7	C__11900619_10	G	A	0.201
4	134859217	rs10029269	CTD-2012I17	survival	3.61×10^{-6}	[2]2	C__30565284_10	G	T	0.227
4	144566782	rs6537296	HHIP	COPD	0.001	[8]8	C___2968767_10	NA	NA	NA
5	1286401	rs2736100	TERT	risk	2.0×10^{-6}	[9]9	C___1844009_10	NA	NA	NA
5	1320607	rs402710	CLPTM1L	survival	0.012	[10]10	C___1150769_20	T	C	0.304
5	1321972	rs401681	CLPTM1L	risk	7.90×10^{-9}	[11]11	C___1150767_20	T	C	0.425
5	157505368	rs2277027	ADAM19	COPD	9.93×10^{-11}	[12]12	C__15883185_10	C	A	0.366
5	177093242	rs351855	FGFR4	survival	0.008	[13]13	C___3166614_10	T	C	0.300
6	28808340	rs4324798	NA	survival	0.001	[14]14	C___2458070_10	A	G	0.038
6	31652743	rs3117582	BAT3	risk	4.97×10^{-10}	[11]11	C__30607439_20	NA	NA	NA
6	31753256	rs3131379	MSH5	risk	1.5×10^{-13}	[15]15	C__25630744_10	T	C	0.047
6	34956087	rs847845	ANKS1A	survival	5.82×10^{-6}	[2]2	C___1257536_10	A	G	0.154
6	169296169	rs7452888	VTA1P1	survival	0.024	[10]10	C__28952867_10	G	A	0.487
7	24943836	rs2710994	OSBPL3	survival	0.008	[10]10	C__15920819_10	T	C	0.162
7	31472538	rs11773530	CCDC129	risk	0.0164	[1]1	C___2573863_10	A	G	0.140
7	85713991	rs4330610	NA	risk	0.009	[1]1	C__27931891_10	T	C	0.028
8	14817078	rs17120323	SGCZ	risk	0.0011	[1]1	C__33892665_10	G	A	0.229
8	70255759	rs2926702	NCOA2	survival	7.33×10^{-6}	[2]2	C___1685982_10	G	A	0.127
8	117013406	rs3019885	SLC30A8	risk	0.021	[1]1	C__26137656_10	G	T	0.449
9	18930224	rs1571228	FAM154A	survival	2.03×10^{-7}	[2]	C___8769215_10	G	A	0.152

Chr. [1]	Map position (bp) [1]	SNP	Closest gene	Original publication			TaqMan assay [2]	Minor allele [3]	Common allele [3]	MAF [3]
				Role	p-value	Reference				
9	22083405	rs1333040	CDKN2B-AS1	risk	2.3×10^{-8}	[15]	C___8766795_10	C	T	0.339
9	96335245	rs6479272	SLC35D2	survival	9.47×10^{-6}	[2]	C___2703455_10	T	C	0.418
10	3782241	rs3750861	KLF6	risk	0.007	[16]	C__25605247_10	T	C	0.085
10	88967577	rs1926203	STAMBPL1	risk	1.28×10^{-6}	[17]	C__12123925_10	G	T	0.358
11	6238271	rs325702	CNGA4	risk	0.0045	[1]	C____543340_10	T	C	0.094
11	38168318	rs820900	NA	risk	0.0114	[1]	C___7551776_10	A	G	0.040
12	955272	rs10849605	RAD52	risk	5.0×10^{-7}	[15]	C___1244798_10	C	T	0.490
12	23524758	rs11046966	SOX5	COPD	1.7×10^{-7}	[18]	C__31578395_10	C	T	0.300
12	31766254	rs6488007	AMN1	risk	0.0192	[7]	C__26622457_10	NA	NA	NA
12	68925008	rs11833102	CPM	risk	0.0006	[1]	C___3191357_10	T	G	0.120
13	91792975	rs2352028	GPC5	risk	5.94×10^{-6}	[19]	C___3029792_10	T	C	0.295
13	101398739	rs9557635	NALCN	survival	8.72×10^{-6}	[2]	C___1806025_10	A	G	0.379
14	74899026	rs732765	DLST	survival	6.51×10^{-6}	[2]	C___1092725_10	G	A	0.193
14	78068563	rs3850370	NA	survival	4.22×10^{-6}	[4]	C___7575699_10	G	T	0.207
15	43262028	rs504417	TGM5	risk	1.2×10^{-6}	[15]	C___2908335_10	G	A	0.378
15	78448622	rs2568494	IREB2	COPD	6.39×10^{-6}	[20]	C__16043098_10	A	G	0.439
15	78565554	rs503464	CHRNA5	risk	NA *	[21]	custom_AH89ZP5	NA	NA	NA
15	78565644	rs55781567	CHRNA5	risk	NA *	[21]	custom_AHABEVY	NA	NA	NA
15	78590583	rs16969968	CHRNA5	risk,<br>survival,<br>COPD	1.00×10^{-20}<br>0.041<br>0.01343	[10, 22, 23, 23]	C__26000428_20	A	G	0.448
15	78596058	rs578776	CHRNA3	risk	0.001	[24]	C____721253_10	T	C	0.232
15	78622903	rs6495309	CHRNB4	risk,<br>survival,<br>COPD	1.1×10^{-32}<br>0.0001<br>0.005	[15, 25, 26, 26]	C__30730895_10	T	C	0.196
15	78625057	rs1948	CHRNB4	Nicotine dependence	0.0071	[27]	C__11941837_10	T	C	0.281
15	78652376	rs11634351	CHRNB4	Nicotine dependence	0.0011	[27]	C____197298_10	A	G	0.454
19	348743	rs6510725	MIER2	survival	2.75×10^{-6}	[2]	C__30955589_20	T	C	0.427
19	32544943	rs1862214	PDCD5	risk,<br>survival	0.0004<br>0.003	[28]	C___2882438_10	G	C	0.235
19	40852719	rs4105144	CYP2A6	risk	0.005	[24]	C__43815914_10	T	C	0.376
19	45405792	rs1005165	PPP1R13L	survival	0.005	[29]	C___2532945_10	T	C	0.166

Chr. [1]	Map position (bp) [1]	SNP	Closest gene	Original publication			TaqMan assay [2]	Minor allele [3]	Common allele [3]	MAF [3]
				Role	p-value	Reference				
19	45406676	rs967591	CD3EAP	survival	0.0001	[29]	C___8713992_1_	A	G	0.170
19	45408744	rs735482	CD3EAP	survival	0.01	[29]	C____341729_10	C	A	0.150
20	23975141	rs755032	GGTLC1	risk	0.0067	[1]	C____611011_10	T	C	0.103
20	46971641	rs13041757	EYA2	survival	6.08×10^{-6}	[3]	C___1274836_10	A	G	0.440
22	21009216	rs2516542	P2RX6	risk	0.0076	[1]	C___2615081_10	T	C	0.070
22	43900170	rs4823153	PNPLA5	risk	0.0046	[1]	custom_AH701JX	G	A	0.277

[1] Chr., chromosome, and map position based on genome assembly GRCh38.p5. [2] TaqMan assay ID (Thermo Fisher Scientific). [3] Minor allele A1, major allele A2, and minor allele frequency (MAF) observed in our series; MAF calculated using PLINK.
NA, not available; COPD, chronic obstructive pulmonary disease
* For these SNPs, association with lung cancer risk was attributed on the basis of their strong linkage disequilibrium with other lung cancer risk associated variants (e.g. rs16969968).

References

1. Galvan, A. *et al*. Genome-wide association study in discordant sibships identifies multiple inherited susceptibility alleles linked to lung cancer. *Carcinogenesis* **31**, 462-465 (2010).

2. Lee, Y. *et al*. Prognostic implications of genetic variants in advanced non-small cell lung cancer: a genome-wide association study. *Carcinogenesis* **34**, 307-313 (2013).

3. Huang, Y. T. *et al*. Genome-wide analysis of survival in early-stage non-small-cell lung cancer. *J. Clin. Oncol.* **27**, 2660-2667 (2009).

4. Hu, L. *et al*. Genome-wide association study of prognosis in advanced non-small cell lung cancer patients receiving platinum-based chemotherapy. *Clin. Cancer Res.* **18**, 5507-5514 (2012).

5. Amos, C. I. *et al*. Genome-wide association scan of tag SNPs identifies a susceptibility locus for lung cancer at 15q25.1. *Nat. Genet.* **40**, 616-622 (2008).

6. Chikova, A., Bernard, H. U., Shchepotin, I. B. & Grando, S. A. New associations of the genetic polymorphisms in nicotinic receptor genes with the risk of lung cancer. *Life Sci.* **91**, 1103-1108 (2012).

7. Galvan, A. *et al*. A polygenic model with common variants may predict lung adenocarcinoma risk in humans. *Int. J. Cancer* **123**, 2327-2330 (2008).

8. Zhou, X. *et al*. Identification of a chronic obstructive pulmonary disease genetic determinant that regulates HHIP. *Hum. Mol. Genet.* **21**, 1325-1335 (2012).

9. McKay, J. D. *et al*. Lung cancer susceptibility locus at 5p15.33. *Nat. Genet.* **40**, 1404-1406 (2008).

10. Xun, W. W. *et al*. Single-nucleotide polymorphisms (5p15.33, 15q25.1, 6p22.1, 6q27 and 7p15.3) and lung cancer survival in the European Prospective Investigation into Cancer and Nutrition (EPIC). *Mutagenesis* **26**, 657-666 (2011).

11. Wang, Y. *et al*. Common 5p15.33 and 6p21.33 variants influence lung cancer risk. *Nat. Genet.* **40**, 1407-1409 (2008).

12. Hancock, D. B. *et al*. Meta-analyses of genome-wide association studies identify multiple loci associated with pulmonary function. *Nat. Genet.* **42**, 45-52 (2010).

13. Spinola, M. *et al*. Functional FGFR4 Gly388Arg polymorphism predicts prognosis in lung adenocarcinoma patients. *J. Clin. Oncol.* **23**, 7307-7311 (2005).

14. Yang, P. *et al*. A rigorous and comprehensive validation: common genetic variations and lung cancer. *Cancer Epidemiol. Biomarkers Prev.* **19**, 240-244 (2010).

15. Timofeeva, M. N. *et al*. Influence of common genetic variation on lung cancer risk: meta-analysis of 14 900 cases and 29 485 controls. *Hum. Mol. Genet.* **21**, 4980-4995 (2012).

16. Spinola, M. *et al*. Genome-wide single nucleotide polymorphism analysis of lung cancer risk detects the KLF6 gene. *Cancer Lett.* **251**, 311-316 (2007).

17. Broderick, P. *et al*. Deciphering the impact of common genetic variation on lung cancer risk: a genome-wide association study. *Cancer Res.* **69**, 6633-6641 (2009).

18. Hersh, C. P. *et al*. SOX5 is a candidate gene for chronic obstructive pulmonary disease susceptibility and is necessary for lung development. *Am. J. Respir. Crit. Care Med.* **183**, 1482-1489 (2011).

19. Li, Y. *et al*. Genetic variants and risk of lung cancer in never smokers: a genome-wide association study. *Lancet Oncol.* **11**, 321-330 (2010).

20. DeMeo, D. L. *et al*. Integration of genomic and genetic approaches implicates IREB2 as a COPD susceptibility gene. *Am. J. Hum. Genet.* **85**, 493-502 (2009).

21. Falvella, F. S. *et al*. Promoter polymorphisms and transcript levels of nicotinic receptor CHRNA5. *J. Natl. Cancer Inst.* **102**, 1366-1370 (2010).

22. Hung, R. J. *et al*. A susceptibility locus for lung cancer maps to nicotinic acetylcholine receptor subunit genes on 15q25. *Nature* **452**, 633-637 (2008).

23. Saccone, N. L. *et al*. Multiple independent loci at chromosome 15q25.1 affect smoking quantity: a meta-analysis and comparison with lung cancer and COPD. *PLoS Genet.* **6**, e1001053 (2010).

24. Timofeeva, M. N. *et al*. Genetic polymorphisms in 15q25 and 19q13 loci, cotinine levels, and risk of lung cancer in EPIC. *Cancer Epidemiol. Biomarkers Prev.* **20**, 2250-2261 (2011).

25. Jin, G. *et al*. A functional polymorphism on chromosome 15q25 associated with survival of early stage non-small-cell lung cancer. *J. Thorac. Oncol.* **7**, 808-814 (2012).

26. Cui, K., Ge, X. & Ma, H. Four SNPs in the CHRNA3/5 alpha-neuronal nicotinic acetylcholine receptor subunit locus are associated with COPD risk based on meta-analyses. *PLoS One* **9**, e102324 (2014).

27. Broms, U. *et al*. Analysis of detailed phenotype profiles reveals CHRNA5-CHRNA3-CHRNB4 gene cluster association with several nicotine dependence traits. *Nicotine Tob. Res.* **14**, 720-733 (2012).

28. Spinola, M. *et al*. Association of the PDCD5 locus with lung cancer risk and prognosis in smokers. *J. Clin. Oncol.* **24**, 1672-1678 (2006).

29. Jeon, H. S. *et al*. A functional variant at 19q13.3, rs967591G>A, is associated with shorter survival of early-stage lung cancer. *Clin. Cancer Res.* **19**, 4185-4195 (2013).

Supplementary Table S2. Ten *cis*-eQTLs and their same-chromosome target genes identified in non-involved lung tissue from 232 lung adenocarcinoma patients (analysis on all 56 SNPs). eQTLs clustered into three main loci

eQTL	SNP	Chr.	Position (bp) [1]	Target gene	Nominal *P*	FDR	Beta [2]
1	rs504417	15	43,262,028	*ADAL*	0.000711	0.011	-0.063
2	rs2568494	15	78,448,622	*IREB2*	1.35×10^{-6}	0.00012	-0.21
3	rs16969968	15	78,590,583	*IREB2*	5.70×10^{-6}	0.00025	-0.20
4	rs578776	15	78,596,058	*PSMA4*	9.69×10^{-5}	0.0029	0.092
5	rs578776	15	78,596,058	*IREB2*	0.000628	0.011	0.17
6	rs6495309	15	78,622,903	*PSMA4*	0.00100	0.013	0.092
7	rs1005165	19	45,405,792	*ERCC1*	0.00175	0.018	-0.10
8	rs967591	19	45,406,676	*VASP*	0.00184	0.018	-0.14
9	rs967591	19	45,406,676	*ERCC1*	0.00394	0.035	-0.092
10	rs735482	19	45,408,744	*ERCC1*	0.000711	0.011	-0.12

[1] Map position based on genome assembly GRCh38.p5. [2] Beta values are from the additive linear regression model (in MatrixEQTL R package), where genotype (expressed as the minor allele count) is assumed to have an additive effect on gene expression. Patients' sex and age at surgery were used as covariates. A positive value indicates that the expression of the target gene increases with an increase in minor allele count.

Supplementary Table S3. PCR primers used for amplification and genotyping of three single nucleotide polymorphisms located within the transcribed region of *IREB2*, *PSMA4*, and *ERCC1* genes for differential allelic expression analysis

Gene	SNP	PCR	Forward primer	Reverse primer	Pyrosequencing primer
IREB2	rs12899351	1	TCATTTATCACCTGCGATGC	TGTGCCTCTGCCCATAACTA	
		2	CGGGTTCAGGGGATTCTC	CGGATCACGAGGTCAAGAGA*	ATGGGGTTTCACCAT
PSMA4	rs8053	1	ATGACCTTGAAGTCAGCACTTG	TGGTGCCCCAAATGAGTAAT	
		2	CAAAAAGAAGTGGAGCAGTTGAT*	TCTTTCTTCTCACGCTCAGCT	TTTGGCTTCTTCTTCC
ERCC1	rs11615	1	ACCCTGGGAAGGACAAAGAG	CTCTGGCCCAGCACATAGTC	
		2	CAATCCCGTACTGAAGTTCGTG*	CTCTGGCCCAGCACATAGTC	CCAAATTCCCAGGGC

* 5’-biotinylated primer. 1: First amplification step. 2: Second amplification step and genotyping.

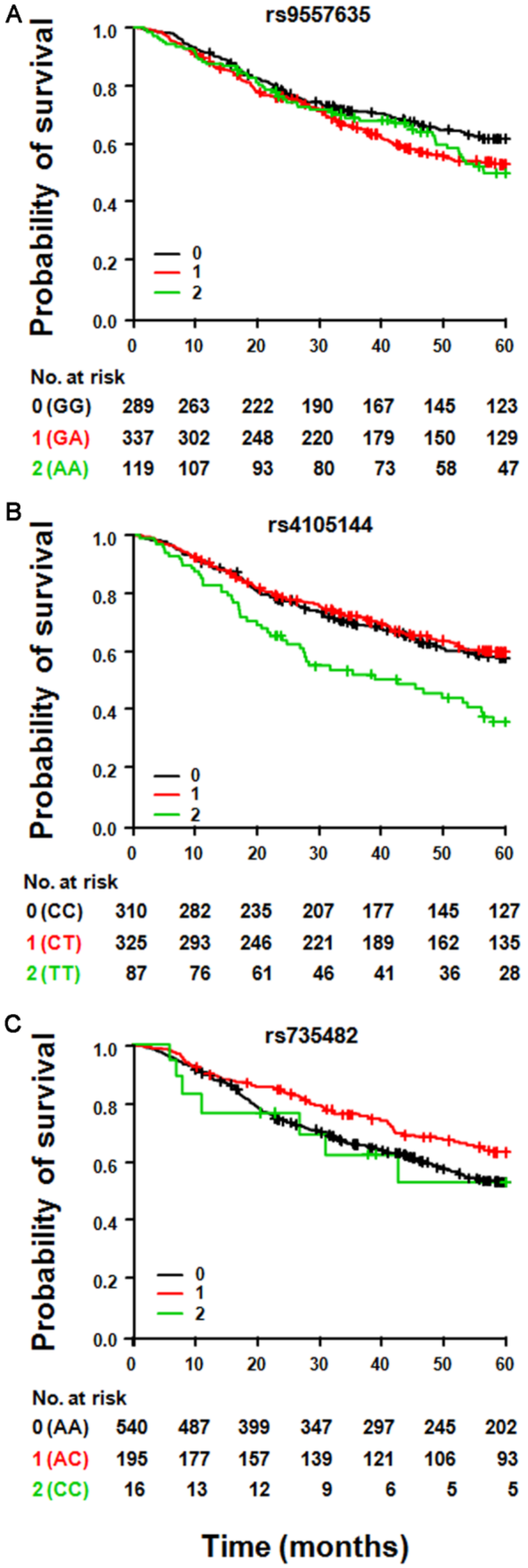
A
rs9557635
Probability of survival
1.0
0.8
0.6
0.4
0.2
0.0
0
10
20
30
40
50
60
0
1
2
No. at risk
0 (GG) 289 263 222 190 167 145 123
1 (GA) 337 302 248 220 179 150 129
2 (AA) 119 107 93 80 73 58 47
B
rs4105144
Probability of survival
No. at risk
0 (CC) 310 282 235 207 177 145 127
1 (CT) 325 293 246 221 189 162 135
2 (TT) 87 76 61 46 41 36 28
C
rs735482
Probability of survival
No. at risk
0 (AA) 540 487 399 347 297 245 202
1 (AC) 195 177 157 139 121 106 93
2 (CC) 16 13 12 9 6 5 5
Time (months)

Supplementary Figure S1. Kaplan-Meier survival curves for lung adenocarcinoma patients, according to the genotypes at three SNPs associated with overall survival. The homozygosity carrier status of the minor allele is shown with green lines, the homozygosity carrier status of the common allele is shown in black, and heterozygosity is shown in red. Crosses denote censored samples. Below the figures are reported the number of patients at risk at the specified times of follow-up.
